# [2-Butyl-4-(4-*tert*-butyl­benz­yl)-1,2,4-triazol-3-yl­idene]chlorido[(1,2,5,6-η)-cyclo­octa-1,5-diene]iridium(I)

**DOI:** 10.1107/S1600536812000992

**Published:** 2012-01-14

**Authors:** Gary S. Nichol, David P. Walton, Laura J. Anna, Edward Rajaseelan

**Affiliations:** a Department of Chemistry & Biochemistry, The University of Arizona, Tucson, AZ 85716, USA; b Department of Chemistry, Millersville University, Millersville, PA 17551, USA

## Abstract

In the title compound, [IrCl(C_8_H_12_)(C_17_H_25_N_3_)], the Ir^I^ ion has a distorted square-planar coordination geometry. The *N*-heterocyclic carbene ligand has an extended S-shaped conformation. The butyl group was refined using a two-part 1:1 disorder model. In the crystal, three unique weak C—H⋯Cl contacts are present. Two of these form a motif described as *R*_2_^1^(6) in graph-set notation, while a third forms an *R*_2_^2^(8) motif about a crystallographic inversion center. The result is a chain structure which extends parallel to the crystallographic *a* axis.

## Related literature

For steric and electronic effects in related *N*-heterocyclic carbene (NHC) ligands, see: Gusev (2009 ▶). For the synthesis, structures and dynamics of related NHC rhodium and iridium complexes, see: Köcher & Herrmann (1997 ▶); Wang & Lin (1998 ▶); Chianese *et al.* (2004 ▶); Herrmann *et al.* (2006 ▶); Nichol *et al.* (2009 ▶, 2010 ▶, 2011 ▶); Lu *et al.* (2011 ▶); Huttenstine *et al.* (2011 ▶). For the catalytic activity of related complexes, see: Hillier *et al.* (2001 ▶); Albrecht *et al.* (2002 ▶); Gnanamgari *et al.* (2007 ▶).
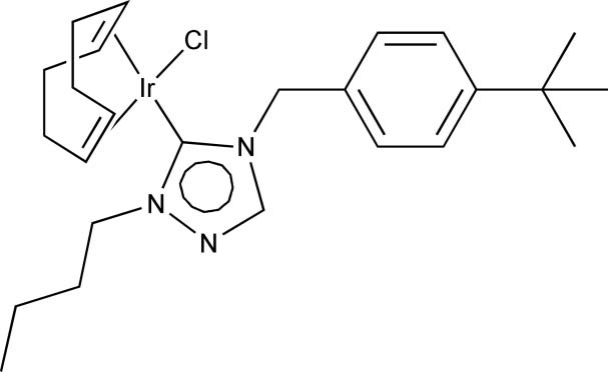


## Experimental

### 

#### Crystal data

[IrCl(C_8_H_12_)(C_17_H_25_N_3_)]
*M* *_r_* = 607.23
Triclinic, 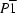

*a* = 10.2485 (3) Å
*b* = 11.2843 (3) Å
*c* = 11.9237 (4) Å
α = 65.213 (2)°
β = 75.170 (2)°
γ = 76.052 (2)°
*V* = 1196.07 (6) Å^3^
*Z* = 2
Mo *K*α radiation
μ = 5.71 mm^−1^
*T* = 100 K
0.30 × 0.10 × 0.06 mm

#### Data collection

Bruker Kappa APEXII DUO CCD diffractometer
Absorption correction: numerical (*SADABS*; Sheldrick, 1996 ▶) *T*_min_ = 0.277, *T*_max_ = 0.744
23768 measured reflections
6993 independent reflections
6168 reflections with *I* > 2σ(*I*)
*R*_int_ = 0.039

#### Refinement

*R*[*F* ^2^ > 2σ(*F* ^2^)] = 0.025
*wR*(*F* ^2^) = 0.059
*S* = 1.03
6993 reflections
313 parameters
106 restraints
H-atom parameters constrained
Δρ_max_ = 1.33 e Å^−3^
Δρ_min_ = −1.83 e Å^−3^

### 

Data collection: *APEX2* (Bruker, 2007 ▶); cell refinement: *SAINT* (Bruker, 2007 ▶); data reduction: *SAINT*; program(s) used to solve structure: *SHELXTL* (Sheldrick, 2008 ▶); program(s) used to refine structure: *SHELXTL*; molecular graphics: *SHELXTL* and *Mercury* (Macrae *et al.*, 2006 ▶); software used to prepare material for publication: *SHELXTL* and *publCIF* (Westrip, 2010 ▶).

## Supplementary Material

Crystal structure: contains datablock(s) I, global. DOI: 10.1107/S1600536812000992/fj2494sup1.cif

Structure factors: contains datablock(s) I. DOI: 10.1107/S1600536812000992/fj2494Isup2.hkl

Supplementary material file. DOI: 10.1107/S1600536812000992/fj2494Isup3.cdx

Additional supplementary materials:  crystallographic information; 3D view; checkCIF report

## Figures and Tables

**Table 1:** Hydrogen-bond geometry (Å, °)

*D*—H⋯*A*	*D*—H	H⋯*A*	*D*⋯*A*	*D*—H⋯*A*
C2—H2⋯Cl1^i^	0.95	2.84	3.568 (3)	135
C7—H7*A*⋯Cl1^i^	0.99	2.77	3.641 (3)	147
C18—H18⋯Cl1^ii^	1.00	2.74	3.617 (3)	147

Symmetry codes: (i) 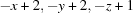; (ii) 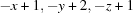.

**Fig. 1.**
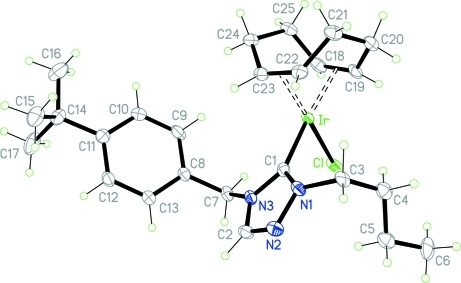
The structure of (I) with displacement ellipsoids at the 50% probability level. The minor disorder component is omitted.

**Fig. 2.**
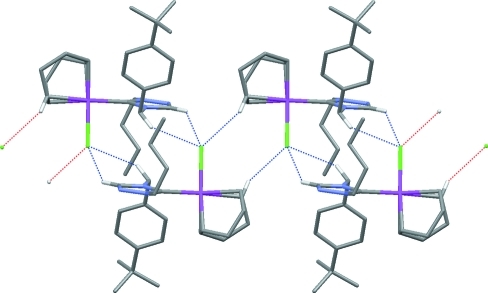
C–H···Cl contacts in (I), shown by blue dotted lines.

## supplementary crystallographic information

### Comment

The title compound, (I), was prepared as part of our ongoing research into complexes of rhodium and iridium with N-heterocyclic carbene (NHC) ligands derived from 1,2,3-triazole (Nichol *et al.*, 2009, 2010, 2011). The Ir center has an expected square planar conformation (Figure 1). The butyl group of the NHC ligand was refined using a two part disorder model, and both disorder components adopt an extended conformation. Three weak C–H···Cl contacts are present (Figure 2, Table 1). Two of these, involving H2 and H7A, combine to form a motif described in graph-set notion as *R*^1^_2_(6), while the third involves H18 and a symmetry related molecule to form an *R*^2^_2_(8) motif, about an inversion center. The combination of these two motifs is a chain structure, connected by weak C–H···Cl contacts, which extends parallel to the crystallographic *a* axis.

### Experimental

Unless otherwise stated, all chemicals were purchased from Sigma Aldrich and Strem and used without further purification, in the dark, and under a nitrogen atmosphere. 1-Butyl-1,2,4-triazole (2.0 g, 16 mmol) and 4-*tert-*butylbenzyl bromide (4.10 g, 18.0 mmol) were refluxed in toluene (15 ml) for 3 d. After cooling, ether (50 ml) was added and the white solid that formed was filtered, washed with ether and air dried (79%). Transmetalation in CH_2_Cl1_2_ (10 ml) with 1-butyl-4-(4-*tert*-butylbenzyl)-1,2,4-triazolium bromide (0.166 g, 0.471 mmol), Ag_2_O (0.0565 g, 0.244 mmol), and [Ir(cod)Cl1]_2_ (0.1585 g, 0.236 mmol), gave a bright yellow solid (95%). X-ray quality crystals were grown from CH_2_Cl1_2_/Pentanes by slow diffusion.

### Refinement

H atoms were initially located in a difference Fourier map and then refined with constrained C–H distances and *U*_iso_(H)=1.5Ueq(C) for methyl H atoms, and *U*_iso_(H)=1.2Ueq(C) for all other H atoms. C atoms C3 to C6 of the butyl chain were refined using a two-part disorder model with a refined major:minor occupancy ratio of approximately 1:1. Similarity restraints were used on the displacement ellipsoids of the disordered components.

### Crystal data

**Table:**

[IrCl(C_8_H_12_)(C_17_H_25_N_3_)]	*Z* = 2
*M**_r_* = 607.23	*F*(000) = 604
Triclinic, *P*1	*D*_x_ = 1.686 Mg m^−^^3^
Hall symbol: -P 1	Mo *K*α radiation, λ = 0.71073 Å
*a* = 10.2485 (3) Å	Cell parameters from 9406 reflections
*b* = 11.2843 (3) Å	θ = 2.5–30.5°
*c* = 11.9237 (4) Å	µ = 5.71 mm^−^^1^
α = 65.213 (2)°	*T* = 100 K
β = 75.170 (2)°	Blade, yellow
γ = 76.052 (2)°	0.30 × 0.10 × 0.06 mm
*V* = 1196.07 (6) Å^3^	

### Data collection

**Table:**

Bruker Kappa APEXII DUO CCD diffractometer	6993 independent reflections
Radiation source: fine-focus sealed tube with Miracol optics	6168 reflections with *I* > 2σ(*I*)
graphite	*R*_int_ = 0.039
thin–slice ω scans	θ_max_ = 30.0°, θ_min_ = 1.9°
Absorption correction: numerical (*SADABS*; Sheldrick, 1996)	*h* = −14→14
*T*_min_ = 0.277, *T*_max_ = 0.744	*k* = −15→15
23768 measured reflections	*l* = −16→16

### Refinement

**Table:**

Refinement on *F*^2^	Primary atom site location: heavy-atom method
Least-squares matrix: full	Secondary atom site location: difference Fourier map
*R*[*F*^2^ > 2σ(*F*^2^)] = 0.025	Hydrogen site location: inferred from neighbouring sites
*wR*(*F*^2^) = 0.059	H-atom parameters constrained
*S* = 1.03	*w* = 1/[σ^2^(*F*_o_^2^) + (0.0323*P*)^2^] where *P* = (*F*_o_^2^ + 2*F*_c_^2^)/3
6993 reflections	(Δ/σ)_max_ = 0.002
313 parameters	Δρ_max_ = 1.33 e Å^−^^3^
106 restraints	Δρ_min_ = −1.83 e Å^−^^3^

### Special details

**Table:**

Geometry. All e.s.d.'s (except the e.s.d. in the dihedral angle between two l.s. planes) are estimated using the full covariance matrix. The cell e.s.d.'s are taken into account individually in the estimation of e.s.d.'s in distances, angles and torsion angles; correlations between e.s.d.'s in cell parameters are only used when they are defined by crystal symmetry. An approximate (isotropic) treatment of cell e.s.d.'s is used for estimating e.s.d.'s involving l.s. planes.
Refinement. Refinement of *F*^2^ against ALL reflections. The weighted *R*-factor *wR* and goodness of fit *S* are based on *F*^2^, conventional *R*-factors *R* are based on *F*, with *F* set to zero for negative *F*^2^. The threshold expression of *F*^2^ > σ(*F*^2^) is used only for calculating *R*-factors(gt) *etc*. and is not relevant to the choice of reflections for refinement. *R*-factors based on *F*^2^ are statistically about twice as large as those based on *F*, and *R*- factors based on ALL data will be even larger.

### Fractional atomic coordinates and isotropic or equivalent isotropic displacement parameters (Å^2^)

**Table:**

	*x*	*y*	*z*	*U*_iso_*/*U*_eq_	Occ. (<1)
Ir1	0.761759 (10)	0.744995 (10)	0.533869 (10)	0.01659 (4)	
Cl1	0.69986 (7)	0.94124 (7)	0.57356 (8)	0.02660 (16)	
N1	1.0046 (2)	0.6417 (2)	0.6693 (2)	0.0195 (5)	
N2	1.1331 (3)	0.6607 (3)	0.6683 (3)	0.0229 (5)	
N3	1.0507 (2)	0.8038 (2)	0.4997 (2)	0.0175 (5)	
C1	0.9505 (3)	0.7272 (3)	0.5684 (3)	0.0173 (5)	
C2	1.1573 (3)	0.7598 (3)	0.5638 (3)	0.0217 (6)	
H2	1.2391	0.7974	0.5349	0.026*	
C3	0.939 (3)	0.547 (2)	0.792 (3)	0.022 (3)	0.496 (11)
H3A	1.0092	0.4931	0.8441	0.026*	0.496 (11)
H3B	0.8961	0.4873	0.7761	0.026*	0.496 (11)
C4	0.8313 (8)	0.6212 (7)	0.8622 (6)	0.0241 (17)	0.496 (11)
H4A	0.7892	0.5561	0.9414	0.029*	0.496 (11)
H4B	0.7589	0.6708	0.8112	0.029*	0.496 (11)
C5	0.8829 (10)	0.7172 (8)	0.8936 (8)	0.0272 (16)	0.496 (11)
H5A	0.9222	0.7847	0.8148	0.033*	0.496 (11)
H5B	0.9565	0.6687	0.9435	0.033*	0.496 (11)
C6	0.770 (2)	0.7856 (18)	0.9672 (19)	0.035 (4)	0.496 (11)
H6A	0.6938	0.8280	0.9212	0.053*	0.496 (11)
H6B	0.8056	0.8526	0.9781	0.053*	0.496 (11)
H6C	0.7383	0.7203	1.0496	0.053*	0.496 (11)
C3'	0.933 (3)	0.551 (3)	0.776 (3)	0.023 (4)	0.504 (11)
H3'1	0.9867	0.4612	0.7964	0.028*	0.504 (11)
H3'2	0.8447	0.5481	0.7580	0.028*	0.504 (11)
C4'	0.9051 (8)	0.5896 (7)	0.8904 (6)	0.0251 (17)	0.504 (11)
H4'1	0.8552	0.5239	0.9632	0.030*	0.504 (11)
H4'2	0.9936	0.5863	0.9115	0.030*	0.504 (11)
C5'	0.8240 (11)	0.7245 (8)	0.8698 (8)	0.0318 (18)	0.504 (11)
H5'1	0.7400	0.7317	0.8395	0.038*	0.504 (11)
H5'2	0.8784	0.7915	0.8040	0.038*	0.504 (11)
C6'	0.785 (2)	0.7534 (17)	0.9910 (18)	0.031 (3)	0.504 (11)
H6'1	0.7287	0.6887	1.0557	0.047*	0.504 (11)
H6'2	0.7324	0.8424	0.9734	0.047*	0.504 (11)
H6'3	0.8675	0.7476	1.0207	0.047*	0.504 (11)
C7	1.0412 (3)	0.9194 (3)	0.3815 (3)	0.0192 (5)	
H7A	1.0807	0.9903	0.3833	0.023*	
H7B	0.9437	0.9527	0.3748	0.023*	
C8	1.1136 (3)	0.8893 (3)	0.2679 (3)	0.0187 (5)	
C9	1.0406 (3)	0.8707 (3)	0.1949 (3)	0.0249 (6)	
H9	0.9440	0.8773	0.2172	0.030*	
C10	1.1065 (3)	0.8425 (3)	0.0896 (3)	0.0251 (6)	
H10	1.0541	0.8295	0.0417	0.030*	
C11	1.2472 (3)	0.8329 (3)	0.0530 (3)	0.0209 (6)	
C12	1.3200 (3)	0.8524 (3)	0.1274 (3)	0.0224 (6)	
H12	1.4167	0.8457	0.1055	0.027*	
C13	1.2544 (3)	0.8812 (3)	0.2317 (3)	0.0219 (6)	
H13	1.3063	0.8956	0.2792	0.026*	
C14	1.3230 (4)	0.8040 (3)	−0.0626 (3)	0.0255 (6)	
C15	1.4246 (4)	0.6764 (4)	−0.0236 (3)	0.0339 (8)	
H15A	1.4865	0.6841	0.0226	0.051*	
H15B	1.4776	0.6610	−0.0986	0.051*	
H15C	1.3749	0.6022	0.0302	0.051*	
C16	1.2278 (4)	0.7869 (4)	−0.1330 (4)	0.0402 (9)	
H16A	1.1774	0.7139	−0.0769	0.060*	
H16B	1.2819	0.7675	−0.2056	0.060*	
H16C	1.1633	0.8685	−0.1616	0.060*	
C17	1.3998 (5)	0.9196 (4)	−0.1525 (3)	0.0412 (10)	
H17A	1.3346	1.0016	−0.1753	0.062*	
H17B	1.4473	0.9032	−0.2284	0.062*	
H17C	1.4666	0.9277	−0.1111	0.062*	
C18	0.5782 (3)	0.7866 (3)	0.4531 (3)	0.0234 (6)	
H18	0.5357	0.8816	0.4244	0.028*	
C19	0.5492 (3)	0.7181 (3)	0.5821 (3)	0.0221 (6)	
H19	0.4886	0.7727	0.6287	0.027*	
C20	0.5410 (3)	0.5724 (3)	0.6472 (3)	0.0262 (6)	
H20A	0.4537	0.5563	0.6389	0.031*	
H20B	0.5410	0.5447	0.7377	0.031*	
C21	0.6599 (3)	0.4876 (3)	0.5934 (3)	0.0255 (6)	
H21A	0.6756	0.3981	0.6588	0.031*	
H21B	0.6355	0.4788	0.5224	0.031*	
C22	0.7903 (3)	0.5482 (3)	0.5485 (3)	0.0207 (6)	
H22	0.8681	0.4900	0.5911	0.025*	
C23	0.8308 (3)	0.6334 (3)	0.4216 (3)	0.0201 (6)	
H23	0.9319	0.6235	0.3919	0.024*	
C24	0.7486 (3)	0.6750 (3)	0.3190 (3)	0.0232 (6)	
H24A	0.7545	0.5989	0.2960	0.028*	
H24B	0.7887	0.7457	0.2437	0.028*	
C25	0.5969 (3)	0.7254 (3)	0.3581 (3)	0.0242 (6)	
H25A	0.5602	0.7918	0.2829	0.029*	
H25B	0.5443	0.6508	0.3943	0.029*	

### Atomic displacement parameters (Å^2^)

**Table:**

	*U*^11^	*U*^22^	*U*^33^	*U*^12^	*U*^13^	*U*^23^
Ir1	0.01197 (5)	0.01520 (5)	0.02372 (6)	−0.00339 (3)	0.00016 (4)	−0.00966 (4)
Cl1	0.0150 (3)	0.0200 (3)	0.0505 (5)	−0.0050 (2)	0.0037 (3)	−0.0230 (3)
N1	0.0157 (11)	0.0181 (11)	0.0243 (13)	−0.0058 (9)	0.0000 (9)	−0.0078 (10)
N2	0.0194 (12)	0.0250 (13)	0.0261 (13)	−0.0042 (10)	−0.0061 (10)	−0.0098 (11)
N3	0.0147 (11)	0.0171 (11)	0.0194 (12)	−0.0035 (9)	0.0006 (9)	−0.0072 (9)
C1	0.0164 (13)	0.0159 (12)	0.0191 (13)	−0.0011 (10)	−0.0006 (10)	−0.0085 (10)
C2	0.0171 (13)	0.0241 (14)	0.0262 (15)	−0.0048 (11)	−0.0042 (11)	−0.0107 (12)
C3	0.028 (6)	0.017 (5)	0.015 (7)	−0.014 (4)	−0.003 (4)	0.004 (4)
C4	0.024 (4)	0.022 (3)	0.022 (3)	−0.006 (3)	−0.004 (3)	−0.002 (2)
C5	0.026 (4)	0.024 (3)	0.029 (4)	−0.008 (3)	−0.004 (3)	−0.006 (3)
C6	0.045 (8)	0.030 (8)	0.019 (7)	−0.014 (6)	0.007 (5)	−0.002 (5)
C3'	0.031 (6)	0.020 (6)	0.017 (7)	−0.003 (4)	−0.006 (4)	−0.005 (5)
C4'	0.027 (4)	0.027 (3)	0.017 (3)	−0.007 (3)	−0.005 (3)	−0.001 (2)
C5'	0.039 (5)	0.028 (3)	0.024 (4)	−0.003 (3)	−0.002 (3)	−0.008 (3)
C6'	0.044 (6)	0.030 (8)	0.018 (7)	−0.006 (6)	−0.007 (5)	−0.008 (6)
C7	0.0166 (13)	0.0160 (12)	0.0207 (14)	−0.0037 (10)	0.0011 (10)	−0.0048 (10)
C8	0.0163 (13)	0.0170 (13)	0.0205 (13)	−0.0040 (10)	−0.0007 (10)	−0.0058 (11)
C9	0.0160 (13)	0.0308 (16)	0.0264 (15)	−0.0095 (12)	−0.0019 (11)	−0.0074 (13)
C10	0.0227 (15)	0.0305 (16)	0.0272 (16)	−0.0087 (12)	−0.0042 (12)	−0.0136 (13)
C11	0.0248 (15)	0.0174 (13)	0.0189 (13)	−0.0062 (11)	−0.0021 (11)	−0.0049 (11)
C12	0.0151 (13)	0.0266 (15)	0.0242 (15)	−0.0039 (11)	−0.0002 (11)	−0.0100 (12)
C13	0.0177 (13)	0.0264 (15)	0.0230 (14)	−0.0061 (11)	−0.0012 (11)	−0.0106 (12)
C14	0.0337 (17)	0.0228 (14)	0.0200 (14)	−0.0059 (13)	−0.0013 (12)	−0.0093 (12)
C15	0.039 (2)	0.0299 (17)	0.0320 (18)	−0.0018 (15)	−0.0023 (15)	−0.0154 (15)
C16	0.049 (2)	0.046 (2)	0.036 (2)	0.0002 (18)	−0.0140 (18)	−0.0261 (18)
C17	0.063 (3)	0.0307 (18)	0.0233 (17)	−0.0152 (18)	0.0110 (17)	−0.0101 (14)
C18	0.0156 (13)	0.0170 (13)	0.0337 (17)	−0.0020 (10)	−0.0051 (12)	−0.0058 (12)
C19	0.0112 (12)	0.0230 (14)	0.0330 (16)	−0.0047 (10)	0.0016 (11)	−0.0135 (12)
C20	0.0234 (15)	0.0273 (16)	0.0294 (16)	−0.0127 (12)	0.0016 (12)	−0.0112 (13)
C21	0.0300 (16)	0.0169 (13)	0.0290 (16)	−0.0079 (12)	−0.0072 (13)	−0.0047 (12)
C22	0.0202 (14)	0.0150 (12)	0.0293 (15)	0.0014 (10)	−0.0062 (11)	−0.0119 (11)
C23	0.0150 (13)	0.0207 (13)	0.0280 (15)	−0.0010 (10)	−0.0036 (11)	−0.0134 (12)
C24	0.0228 (15)	0.0251 (15)	0.0271 (15)	−0.0068 (12)	−0.0046 (12)	−0.0133 (12)
C25	0.0176 (14)	0.0244 (15)	0.0326 (17)	−0.0060 (11)	−0.0075 (12)	−0.0096 (13)

### Geometric parameters (Å, °)

**Table:**

Ir1—Cl1	2.3618 (7)	C8—C13	1.389 (4)
Ir1—C18	2.193 (3)	C9—H9	0.950
Ir1—C19	2.168 (3)	C9—C10	1.389 (5)
Ir1—C22	2.106 (3)	C10—H10	0.950
Ir1—C23	2.092 (3)	C10—C11	1.387 (4)
N1—N2	1.381 (3)	C11—C12	1.405 (4)
N1—C1	1.340 (4)	C11—C14	1.522 (4)
N1—C3	1.51 (3)	C12—H12	0.950
N1—C3'	1.42 (3)	C12—C13	1.382 (4)
N2—C2	1.293 (4)	C13—H13	0.950
N3—C1	1.369 (3)	C14—C15	1.529 (5)
N3—C2	1.367 (4)	C14—C16	1.529 (5)
N3—C7	1.471 (4)	C14—C17	1.536 (5)
C2—H2	0.950	C15—H15A	0.980
C3—H3A	0.990	C15—H15B	0.980
C3—H3B	0.990	C15—H15C	0.980
C3—C4	1.51 (3)	C16—H16A	0.980
C4—H4A	0.990	C16—H16B	0.980
C4—H4B	0.990	C16—H16C	0.980
C4—C5	1.519 (11)	C17—H17A	0.980
C5—H5A	0.990	C17—H17B	0.980
C5—H5B	0.990	C17—H17C	0.980
C5—C6	1.52 (3)	C18—H18	1.000
C6—H6A	0.980	C18—C19	1.390 (5)
C6—H6B	0.980	C18—C25	1.509 (5)
C6—H6C	0.980	C19—H19	1.000
C3'—H3'1	0.990	C19—C20	1.509 (4)
C3'—H3'2	0.990	C20—H20A	0.990
C3'—C4'	1.54 (3)	C20—H20B	0.990
C4'—H4'1	0.990	C20—C21	1.537 (5)
C4'—H4'2	0.990	C21—H21A	0.990
C4'—C5'	1.499 (12)	C21—H21B	0.990
C5'—H5'1	0.990	C21—C22	1.519 (4)
C5'—H5'2	0.990	C22—H22	1.000
C5'—C6'	1.55 (2)	C22—C23	1.426 (4)
C6'—H6'1	0.980	C23—H23	1.000
C6'—H6'2	0.980	C23—C24	1.512 (4)
C6'—H6'3	0.980	C24—H24A	0.990
C7—H7A	0.990	C24—H24B	0.990
C7—H7B	0.990	C24—C25	1.544 (4)
C7—C8	1.500 (4)	C25—H25A	0.990
C8—C9	1.387 (4)	C25—H25B	0.990
			
Cl1—Ir1—C1	88.85 (8)	C9—C10—H10	119.3
Cl1—Ir1—C18	91.89 (8)	C9—C10—C11	121.4 (3)
Cl1—Ir1—C19	90.20 (8)	H10—C10—C11	119.3
Cl1—Ir1—C22	164.65 (9)	C10—C11—C12	117.0 (3)
Cl1—Ir1—C23	155.34 (9)	C10—C11—C14	123.1 (3)
C1—Ir1—C18	167.26 (11)	C12—C11—C14	119.9 (3)
C1—Ir1—C19	155.58 (12)	C11—C12—H12	119.2
C1—Ir1—C22	93.31 (11)	C11—C12—C13	121.5 (3)
C1—Ir1—C23	93.28 (11)	H12—C12—C13	119.2
C18—Ir1—C19	37.16 (12)	C8—C13—C12	120.8 (3)
C18—Ir1—C22	89.33 (11)	C8—C13—H13	119.6
C18—Ir1—C23	80.82 (12)	C12—C13—H13	119.6
C19—Ir1—C22	81.58 (11)	C11—C14—C15	109.6 (3)
C19—Ir1—C23	97.63 (11)	C11—C14—C16	112.9 (3)
C22—Ir1—C23	39.73 (12)	C11—C14—C17	108.7 (3)
N2—N1—C1	113.8 (2)	C15—C14—C16	107.7 (3)
N2—N1—C3	114.9 (12)	C15—C14—C17	109.8 (3)
N2—N1—C3'	121.3 (13)	C16—C14—C17	108.3 (3)
C1—N1—C3	130.1 (12)	C14—C15—H15A	109.5
C1—N1—C3'	124.4 (13)	C14—C15—H15B	109.5
N1—N2—C2	103.3 (2)	C14—C15—H15C	109.5
C1—N3—C2	108.4 (2)	H15A—C15—H15B	109.5
C1—N3—C7	125.7 (2)	H15A—C15—H15C	109.5
C2—N3—C7	125.9 (2)	H15B—C15—H15C	109.5
Ir1—C1—N1	128.0 (2)	C14—C16—H16A	109.5
Ir1—C1—N3	129.1 (2)	C14—C16—H16B	109.5
N1—C1—N3	102.8 (2)	C14—C16—H16C	109.5
N2—C2—N3	111.7 (3)	H16A—C16—H16B	109.5
N2—C2—H2	124.1	H16A—C16—H16C	109.5
N3—C2—H2	124.1	H16B—C16—H16C	109.5
N1—C3—H3A	109.5	C14—C17—H17A	109.5
N1—C3—H3B	109.5	C14—C17—H17B	109.5
N1—C3—C4	110.9 (16)	C14—C17—H17C	109.5
H3A—C3—H3B	108.1	H17A—C17—H17B	109.5
H3A—C3—C4	109.5	H17A—C17—H17C	109.5
H3B—C3—C4	109.5	H17B—C17—H17C	109.5
C3—C4—H4A	108.5	Ir1—C18—H18	114.0
C3—C4—H4B	108.5	Ir1—C18—C19	70.47 (17)
C3—C4—C5	115.1 (12)	Ir1—C18—C25	113.0 (2)
H4A—C4—H4B	107.5	H18—C18—C19	114.0
H4A—C4—C5	108.5	H18—C18—C25	114.0
H4B—C4—C5	108.5	C19—C18—C25	123.8 (3)
C4—C5—H5A	109.1	Ir1—C19—C18	72.37 (17)
C4—C5—H5B	109.1	Ir1—C19—H19	114.0
C4—C5—C6	112.3 (11)	Ir1—C19—C20	108.9 (2)
H5A—C5—H5B	107.9	C18—C19—H19	114.0
H5A—C5—C6	109.1	C18—C19—C20	125.5 (3)
H5B—C5—C6	109.1	H19—C19—C20	114.0
N1—C3'—H3'1	109.4	C19—C20—H20A	109.0
N1—C3'—H3'2	109.4	C19—C20—H20B	109.0
N1—C3'—C4'	111.1 (17)	C19—C20—C21	112.7 (2)
H3'1—C3'—H3'2	108.0	H20A—C20—H20B	107.8
H3'1—C3'—C4'	109.4	H20A—C20—C21	109.0
H3'2—C3'—C4'	109.4	H20B—C20—C21	109.0
C3'—C4'—H4'1	108.8	C20—C21—H21A	109.3
C3'—C4'—H4'2	108.8	C20—C21—H21B	109.3
C3'—C4'—C5'	113.8 (12)	C20—C21—C22	111.5 (2)
H4'1—C4'—H4'2	107.7	H21A—C21—H21B	108.0
H4'1—C4'—C5'	108.8	H21A—C21—C22	109.3
H4'2—C4'—C5'	108.8	H21B—C21—C22	109.3
C4'—C5'—H5'1	109.2	Ir1—C22—C21	114.4 (2)
C4'—C5'—H5'2	109.2	Ir1—C22—H22	114.1
C4'—C5'—C6'	112.1 (10)	Ir1—C22—C23	69.62 (16)
H5'1—C5'—H5'2	107.9	C21—C22—H22	114.1
H5'1—C5'—C6'	109.2	C21—C22—C23	122.9 (3)
H5'2—C5'—C6'	109.2	H22—C22—C23	114.1
C5'—C6'—H6'1	109.5	Ir1—C23—C22	70.66 (16)
C5'—C6'—H6'2	109.5	Ir1—C23—H23	113.9
C5'—C6'—H6'3	109.5	Ir1—C23—C24	113.0 (2)
H6'1—C6'—H6'2	109.5	C22—C23—H23	113.9
H6'1—C6'—H6'3	109.5	C22—C23—C24	124.1 (3)
H6'2—C6'—H6'3	109.5	H23—C23—C24	113.9
N3—C7—H7A	109.0	C23—C24—H24A	109.1
N3—C7—H7B	109.0	C23—C24—H24B	109.1
N3—C7—C8	113.0 (2)	C23—C24—C25	112.7 (3)
H7A—C7—H7B	107.8	H24A—C24—H24B	107.8
H7A—C7—C8	109.0	H24A—C24—C25	109.1
H7B—C7—C8	109.0	H24B—C24—C25	109.1
C7—C8—C9	120.5 (3)	C18—C25—C24	111.8 (2)
C7—C8—C13	121.4 (3)	C18—C25—H25A	109.2
C9—C8—C13	118.1 (3)	C18—C25—H25B	109.2
C8—C9—H9	119.5	C24—C25—H25A	109.2
C8—C9—C10	121.1 (3)	C24—C25—H25B	109.2
H9—C9—C10	119.5	H25A—C25—H25B	107.9
			
C1—N1—N2—C2	−0.3 (3)	C12—C11—C14—C17	−59.3 (4)
C3—N1—N2—C2	−169.4 (11)	Cl1—Ir1—C18—C19	−87.83 (17)
C3'—N1—N2—C2	−172.6 (12)	Cl1—Ir1—C18—C25	152.7 (2)
N2—N1—C1—Ir1	−175.89 (19)	C1—Ir1—C18—C19	179.0 (4)
N2—N1—C1—N3	0.3 (3)	C1—Ir1—C18—C25	59.6 (6)
C3—N1—C1—Ir1	−8.8 (13)	C19—Ir1—C18—C25	−119.4 (3)
C3—N1—C1—N3	167.3 (13)	C22—Ir1—C18—C19	76.87 (19)
C3'—N1—C1—Ir1	−3.9 (13)	C22—Ir1—C18—C25	−42.6 (2)
C3'—N1—C1—N3	172.3 (12)	C23—Ir1—C18—C19	115.85 (19)
C2—N3—C1—Ir1	175.9 (2)	C23—Ir1—C18—C25	−3.6 (2)
C2—N3—C1—N1	−0.2 (3)	Ir1—C18—C19—C20	−100.9 (3)
C7—N3—C1—Ir1	−0.2 (4)	C25—C18—C19—Ir1	105.2 (3)
C7—N3—C1—N1	−176.3 (2)	C25—C18—C19—C20	4.3 (5)
Cl1—Ir1—C1—N1	106.7 (2)	Cl1—Ir1—C19—C18	92.87 (17)
Cl1—Ir1—C1—N3	−68.5 (2)	Cl1—Ir1—C19—C20	−144.7 (2)
C18—Ir1—C1—N1	−159.8 (4)	C1—Ir1—C19—C18	−179.5 (2)
C18—Ir1—C1—N3	25.0 (6)	C1—Ir1—C19—C20	−57.0 (4)
C19—Ir1—C1—N1	18.7 (4)	C18—Ir1—C19—C20	122.4 (3)
C19—Ir1—C1—N3	−156.5 (2)	C22—Ir1—C19—C18	−100.14 (19)
C22—Ir1—C1—N1	−58.1 (3)	C22—Ir1—C19—C20	22.3 (2)
C22—Ir1—C1—N3	126.7 (3)	C23—Ir1—C19—C18	−63.68 (19)
C23—Ir1—C1—N1	−97.9 (3)	C23—Ir1—C19—C20	58.7 (2)
C23—Ir1—C1—N3	86.9 (3)	Ir1—C19—C20—C21	−36.1 (3)
N1—N2—C2—N3	0.2 (3)	C18—C19—C20—C21	45.3 (4)
C1—N3—C2—N2	0.0 (3)	C19—C20—C21—C22	32.9 (4)
C7—N3—C2—N2	176.2 (3)	C20—C21—C22—Ir1	−13.2 (3)
N2—N1—C3—C4	103.3 (17)	C20—C21—C22—C23	−93.8 (3)
C1—N1—C3—C4	−64 (2)	Cl1—Ir1—C22—C21	53.2 (4)
N1—C3—C4—C5	−59 (2)	Cl1—Ir1—C22—C23	171.1 (2)
C3—C4—C5—C6	−178.5 (15)	C1—Ir1—C22—C21	151.0 (2)
N2—N1—C3'—C4'	62 (2)	C1—Ir1—C22—C23	−91.16 (18)
C1—N1—C3'—C4'	−109.9 (17)	C18—Ir1—C22—C21	−41.5 (2)
C3—N1—C3'—C4'	37 (16)	C18—Ir1—C22—C23	76.37 (18)
N1—C3'—C4'—C5'	59 (2)	C19—Ir1—C22—C21	−5.0 (2)
C3'—C4'—C5'—C6'	173.5 (16)	C19—Ir1—C22—C23	112.85 (18)
C1—N3—C7—C8	−101.1 (3)	C23—Ir1—C22—C21	−117.9 (3)
C2—N3—C7—C8	83.4 (3)	Ir1—C22—C23—C24	−105.2 (3)
N3—C7—C8—C9	101.8 (3)	C21—C22—C23—Ir1	106.5 (3)
N3—C7—C8—C13	−79.1 (3)	C21—C22—C23—C24	1.3 (4)
C7—C8—C9—C10	−179.9 (3)	Cl1—Ir1—C23—C22	−174.37 (15)
C13—C8—C9—C10	1.1 (5)	Cl1—Ir1—C23—C24	−54.6 (3)
C8—C9—C10—C11	−0.5 (5)	C1—Ir1—C23—C22	91.22 (18)
C9—C10—C11—C12	0.3 (5)	C1—Ir1—C23—C24	−149.0 (2)
C9—C10—C11—C14	−179.2 (3)	C18—Ir1—C23—C22	−100.14 (18)
C10—C11—C12—C13	−0.7 (5)	C18—Ir1—C23—C24	19.7 (2)
C14—C11—C12—C13	178.8 (3)	C19—Ir1—C23—C22	−66.89 (18)
C11—C12—C13—C8	1.3 (5)	C19—Ir1—C23—C24	52.9 (2)
C7—C8—C13—C12	179.5 (3)	C22—Ir1—C23—C24	119.8 (3)
C9—C8—C13—C12	−1.4 (5)	Ir1—C23—C24—C25	−32.7 (3)
C10—C11—C14—C15	−119.9 (3)	C22—C23—C24—C25	48.9 (4)
C10—C11—C14—C16	0.0 (4)	Ir1—C18—C25—C24	−12.7 (3)
C10—C11—C14—C17	120.1 (4)	C19—C18—C25—C24	−94.0 (3)
C12—C11—C14—C15	60.6 (4)	C23—C24—C25—C18	29.2 (4)
C12—C11—C14—C16	−179.4 (3)		

### Hydrogen-bond geometry (Å, °)

**Table:**

*D*—H···*A*	*D*—H	H···*A*	*D*···*A*	*D*—H···*A*
C2—H2···Cl1^i^	0.95	2.84	3.568 (3)	135
C7—H7A···Cl1^i^	0.99	2.77	3.641 (3)	147
C18—H18···Cl1^ii^	1.00	2.74	3.617 (3)	147

Symmetry codes: (i) −*x*+2, −*y*+2, −*z*+1; (ii) −*x*+1, −*y*+2, −*z*+1.
